# Cyberbullying in Medical Schools: Prevalence, Self-Esteem Implications, Coping Strategies, and the Role of Social Support

**DOI:** 10.7759/cureus.78156

**Published:** 2025-01-28

**Authors:** Namita Deshmukh, Alicia Aranha, Avinash Borkar, Gajanan Velhal

**Affiliations:** 1 Community Medicine, Bhaktshreshtha Kamalakarpant Laxman (BKL) Walawalkar Rural Medical College, Chiplun, IND

**Keywords:** cyberbullying, low self-esteem, mixed methods research, offenders, victimization

## Abstract

Background: Cyberbullying has emerged as a pervasive issue, particularly within educational settings, where its effects can be particularly damaging. Among vulnerable populations, medical students face unique pressures that can exacerbate the impact of cyberbullying. Research indicates that cyberbullying can lead to severe psychological consequences, including anxiety, depression, low self-esteem, and decreased academic performance. The anonymity and reach of digital platforms can facilitate bullying behaviors, leading to a culture of silence and fear. This study was done to explore the nature and scope of cyberbullying and its impact on self-esteem among medical students.

Material and methods: A mixed-method study was carried out among 453 undergraduate medical students at a rural medical college between September 2023 and February 2024. Data were collected using a semi-structured, self-administered questionnaire, which included socio-demographic details, the Cyberbullying Aggressor Scale, and Rosenberg’s Self-Esteem Scale. Focus group discussions (FGDs) were conducted with 56 students who were selected due to a higher prevalence of cyberbullying and its impact on self-esteem. Thematic analysis was used to identify patterns and themes in the qualitative data. Data triangulation was performed to contextualize and explain the quantitative findings through qualitative insights.

Results: Among 453 participants, 154 (34%) were identified as victims of cyberbullying, while 95 (21%) were found to engage in cyberbullying others (offenders). Of the victims, 92 (59.74%) were males and 62 (40.26%) were females. However, among those who cyberbullied others, 87 (91.57%) were males and 8 (8.42%) were females. Low self-esteem was observed in 135 (29.80%) of students, with approximately 110 (72%) of cyberbullying victims reporting lower self-esteem. The fast-paced nature of online interactions was recognized as a factor that can escalate conflicts. Many students noted that misunderstandings and misinterpretations of messages contribute to the occurrence of cyberbullying. Additionally, academic pressure was cited as a significant cause of harmful online interactions, including harassment related to grades or clinical performance.

Conclusion: This study concluded that students who experienced cyberbullying both as victims and offenders exhibited diminished self-esteem. The sociocultural perspective underscored the role of institutional culture and peer dynamics, highlighting the importance of creating a supportive environment that encourages open communication and reduces stigma around seeking help.

## Introduction

In the present era of technological progress, communication relies significantly on digital technology. It’s nearly inconceivable to envision a world without social media or, for that matter, the electronic devices that we own. Online technology usage may give rise to detrimental behaviors, with cyberbullying being one notable example [1]. Bullying involves a deliberate and repetitive effort to harm others, creating a situation where the victim faces difficulty defending themselves due to an imbalance of power. Cyberbullying has been defined as bullying that is perpetrated through the medium of technology, such as on social media or through any form of text message, pictures, video clips, or phone calls [2]. It is mostly seen in young adults. It is becoming prevalent because of its widespread accessibility. Abaido [1] reported a 91% prevalence of cyberbullying, while Lavanya and Prasad [2] found it in just 7% of university students, highlighting a significant variation in its prevalence.

Unlike traditional bullying which requires strong physical built or influential connections, cyberbullying can be done by anyone. Cyberbullying and traditional bullying exhibit three main similarities: they both involve deliberate acts of aggression, occur within relationships characterized by a power imbalance, and commonly manifest as repetitive behaviors [3]. Today’s youth are often seen as digital natives, thriving on social media for social connections and using these platforms for self-discovery and boosting their self-esteem. However, this comes with the risk of exposure to cyberbullying and notable adverse consequences, including anxiety, depression, substance abuse, sleep and eating disorders, and a decline in academic performance [4,5]. Studies have even reported suicidal ideation in the victims of cyberbullying immediately after the episode has occurred. The most significant emotional and non-physical results of cyberbullying include anger, helplessness, grief, and anxiety. Lalitha PD found a positive correlation between cyberbullying and low self-esteem [3,5-7]. Again, many medical colleges located in remote areas lack recreational activities like urban areas, due to which they often engage themselves in social media platforms, becoming easy victims of cyberbullying. Victims are reported to have feelings of being worried, low self-esteem, sleep disturbances, and being irritated with others. Any type of bullying that impacts the self-esteem of a person becomes a health concern that needs extensive research on the subject.

Quantitative methods only measure the extent of its impact, but, qualitative methods explore the personal experiences and perceptions of self-esteem of the subjects in the context of these experiences. Investigating contextual factors like social support, coping strategies, and demographic variables in the interplay of cyberbullying and self-esteem is also an area less explored. Hence, the current study was undertaken with the research question - What are the prevalence rates of cyberbullying victimization and offending and its effect on their self-esteem?

The objectives of the study are as follows: 1. To assess the prevalence of cyberbullying and the degree of self-esteem among medical undergraduates; 2. To assess the impact of cyberbullying on the self-esteem of medical undergraduates; 3. To explore coping strategies against cyberbullying by medical students; 4. To assess the perception of medical undergraduates regarding the role of social support networks in mitigating the effects of cyberbullying.

## Materials and methods

The study was conducted among undergraduate medical students (MBBS) from the 1st to 3rd year using a mixed-method approach, with both quantitative and qualitative components at a medical college in the Konkan region from September 2023 to February 2024. Taking the prevalence of cyberbullying to about 47% [8], the sample size was calculated with a confidence interval of 5% to be 234.

Formula

Population size (for finite population correction factor) (N): 600, Hypothesized % frequency of outcome factor in the population (p):47%+/-5, Confidence limits as % of 100(absolute +/- %) (d): 5%, Design effect (for cluster surveys-DEFF): 1; Sample size n = [DEFF*Np(1-p)]/ [(d2/Z21-α/2*(N-1) + p*(1-p)] = 234. Sampling technique: Convenient sampling.

Methodology

The study was conducted among undergraduate medical students at a rural medical college using a mixed-methods research approach for data collection and analysis. There were 150 students each in the 1st and 2nd phases, and 100 students each in the 3rd and 4th phases. Initially, students from the first two phases were invited to participate after obtaining approval from the Institutional Ethics Committee. However, students from the 3rd and 4th phases also expressed interest and voluntarily joined the study. As a result, a total of 453 students were recruited. All participants were fully informed about the study’s purpose and nature, and their consent was obtained prior to inclusion.

The research was conducted in two phases.

Quantitative Component

Enrolling the participants and collecting information through Google Forms was the first phase of data collection. A preformed, pre-structured, and pre-tested questionnaire was used to inquire about the socio-demographic details of the student’s age, gender, socioeconomic status, academic year of enrollment, and type of 12th board of study. Cyberbullying was assessed by the self-administered cyberbullying aggressor scale and self-esteem was assessed by the self-administered Rosenberg Self-Esteem Scale.

Qualitative Component

After this initial data collection, seven sessions of Focus Group Discussion (FGD) were organized with a total of 56 students selected based on their experience as victims and offenders without declaring the results of the initial assessment. Confidentiality was maintained in this regard in order to facilitate and encourage an open discussion on the sensitive topic of cyberbullying and how it impacted self-esteem, reasons for offenders to indulge in cyberbullying, the coping mechanisms of victims, and the role of social support in dealing with the ill effects of cyberbullying. The participants were divided into seven groups of eight students each. After briefing them about the purpose and method of FGD, their informed written consent was sought. The discussions took place in different classrooms, with a designated trained faculty from the Department of Community Medicine as facilitators. A pre-tested and validated, semi-structured questionnaire with open-ended questions was used for facilitating the FGD. The sessions were video-recorded for further reference. Codes were assigned to meaningful responses and related codes were grouped into themes. Themes were refined through peer and participant reviews. The information gathered was presented in a structured format after a lot of brainstorming and discussions and gaining the consensus of all authors.

Inclusion criteria

Medical undergraduate students of phase I and II not having major medical or surgical problems, a history of psychosis or mania, major depressive disorders, or any other mental disorder. Students from phases III and IV willingly participated in the survey were also included

Exclusion criteria

Students not willing to participate.

Tools

Cyberbullying Aggressor Scale (CYBA) [8]

This includes 32 items, each assessed on a Likert scale of five points. Out of the 32 questions, questions from 1 to 6 and 9 to 11 were related to cyberbullying victimization. This adds up to a total of nine questions in which the responses were scored from 0 to 4. The scores ranged from 0 to 36. The students who scored more than 9 were considered to be victims of cyberbullying whereas individuals who scored less than 9 did not face cyberbullying at all in the past 30 days. Similarly, the questions from 14 to 18 were associated with individuals who cyberbullied others (offenders). The responses to these questions range from 0 to 4 and their score range was 0 to 20. Hence, students with a score of more than 5 were considered to have cyberbullied others in the past 30 days besides them the students who scored less than 5 did not cyberbully anyone. The scale has good reliability and validity for use in the public domain. (Cronbach’s alpha is >0.70) (Table 1) [8].

**Table 1:** Cyberbullying Aggressor Scale (CYBA) [8]

Cyberbullying Aggressor Scale (CYBA)		
Part A - How often in the last 30 days have you experienced the following?		
SN	Question	Response
1	In the last 30 days, have you been made fun of in a chat room?	a. Never b. Once or twice c. A few times d. Many times e. Everyday
2	In the last 30 days, have you received an email from someone you know that made you really mad?	a. Never b. Once or twice c. A few times d. Many times e. Everyday
3	In the last 30 days, have you received an email from someone you didn’t know that made you really mad? This does not include “spam” mail.	a. Never b. Once or twice c. A few times d. Many times e. Everyday
4	In the last 30 days, has someone posted something on your My Space page that made you upset or uncomfortable?	a. Never b. Once or twice c. A few times d. Many times e. Everyday
5	In the last 30 days, has someone posted something on another web page that made you upset or uncomfortable?	a. Never b. Once or twice c. A few times d. Many times e. Everyday
6	In the last 30 days, have you received an instant message that made you upset or uncomfortable?	a. Never b. Once or twice c. A few times d. Many times e. Everyday
7	In the last 30 days, have your parents talked to you about being safe on the computer?	a. Never b. Once or twice c. A few times d. Many times e. Everyday
8	In the last 30 days, has a teacher talked to you about being safe on the computer?	a. Never b. Once or twice c. A few times d. Many times e. Everyday
9	In the last 30 days, have you been bullied or picked on by another person while online?	a. Never b. Once or twice c. A few times d. Many times e. Everyday
10	In the last 30 days, have you been afraid to go on the computer?	a. Never b. Once or twice c. A few times d. Many times e. Everyday
11	In the last 30 days, has anyone posted anything about you online that you didn’t want others to see?	a. Never b. Once or twice c. A few times d. Many times e. Everyday
12	In the last 30 days, has anyone emailed or text messaged you and asked questions about sex that made you uncomfortable?	a. Never b. Once or twice c. A few times d. Many times e. Everyday
Part B - How often in the last 30 days have you done the following?		
13	In the last 30 days, have you lied about your age while online?	a. Never b. Once or twice c. A few times d. Many times e. Everyday
14	In the last 30 days, have you posted something online about someone else to make others laugh?	a. Never b. Once or twice c. A few times d. Many times e. Everyday
15	In the last 30 days, have you sent someone a computer text message to make them angry or to make fun of them?	a. Never b. Once or twice c. A few times d. Many times e. Everyday
16	In the last 30 days, have you sent someone an email to make them angry or to make fun of them?	a. Never b. Once or twice c. A few times d. Many times e. Everyday
17	In the last 30 days, have you posted something on someone’s My Space, Xanga, or Friendster page to make them angry or to make fun of them?	a. Never b. Once or twice c. A few times d. Many times e. Everyday
18	In the last 30 days, have you taken a picture of someone and posted it online without their permission?	a. Never b. Once or twice c. A few times d. Many times e. Everyday
Cyberbullying Aggressor Scale (CYBA) - Part C		
19	In my entire life, I have cyberbullied others	a. Never b. seldom c. sometime d. fairly often e. often f. very often
20	In the last 30 days, I have cyberbullied others	a. Never b. once or twice c. a few times d. many times e. every day
21	If so, what was the most important reason for cyberbullying that person?	a. To get revenge b. they deserved it c. because others were doing it d. for fun e. because they picked on me at school to vent my anger g. to demonstrate power h. I hate them i. other reasons j. I have not cyberbullied another person in the last 30 days
22	In my entire life, I have been cyberbullied	a. Never b. seldom c. sometime d. fairly often e. often f. very often
23	In the last 30 days, I have been cyberbullied	a. Never b. once or twice c. a few times d. many times e. every day
24	Did you know who it was who did this to you?	a. Friend b. someone else from school c. ex-friend d. ex-boyfriend or girlfriend e. someone I knew from a chat room f. stranger g. many people h. other i. No one has ever cyberbullied me
25	Was the bully someone you have met in real life?	a. Yes b. no c. don’t know d. No one has ever cyberbullied me
	How often in the last 30 days have you done the following?	
26	Were you ever cyberbullied by another student at your school?	Never/Once/Sometimes/Often/Many times
27	Were threats made online carried out at school?	Never/Once/Sometimes/Often/Many times
28	Did you tell someone about the cyberbullying experience?	Never/Once/Sometimes/Often/Many times
29	Did you tell your parents about the cyberbullying experience?	Never/Once/Sometimes/Often/Many times
30	Did you tell a friend about the cyberbullying experience?	Never/Once/Sometimes/Often/Many times
31	Did you tell a teacher about the cyberbullying experience?	Never/Once/Sometimes/Often/Many times
32	How did you respond to the cyberbullying experience?	a. logged off computer b. blocked bully c. changed screen name or email d. left site e. called the police f. did nothing g. did something else h. No one has ever cyberbullied me

Rosenberg Self-esteem Scale (RSE) [9]

Each item was rated on a 4-point Likert scale including reverse scoring in some questions. Ten questions related to self-esteem had responses that scored from 1 to 4. The scores obtained ranged from 10 to 40. A high score was related to high self-esteem while a low score indicated low self-esteem. A score of 20 was taken as the cutoff for assessing the prevalence of low self-esteem among the students. Individuals scoring less than equal to 20 were considered to have low self-esteem. The scale has good reliability and validity for use in the public domain. (Cronbach’s alpha >0.77-0.88) (Table 2) [9]. The data was collected using Google Forms and analyzed using IBM SPSS 22. Quantitative data was represented as the mean and standard deviation. Qualitative data was presented as percentages and numbers. Chi-square and Z-test were used for comparison.

**Table 2:** Rosenberg Self-esteem Scale (RSE) [9]

Rosenberg Self-esteem Scale (RSE) Instructions: Below is a list of statements dealing with your general feelings about yourself. Please indicate how strongly you agree or disagree with each statement.		
1	On the whole, I am satisfied with myself.	Strongly disagree/Disagree/Agree/Strongly agree
2	At times I think I am no good at all.	Strongly disagree/Disagree/Agree/Strongly agree
3	I feel that I have a number of good qualities.	Strongly disagree/Disagree/Agree/Strongly agree
4	I am able to do things as well as most other people.	Strongly disagree/Disagree/Agree/Strongly agree
5	I feel I do not have much to be proud of.	Strongly disagree/Disagree/Agree/Strongly agree
6	I certainly feel useless at times.	Strongly disagree/Disagree/Agree/Strongly agree
7	I feel that I'm a person of worth, at least on an equal plane with others.	Strongly disagree/Disagree/Agree/Strongly agree
8	I wish I could have more respect for myself.	Strongly disagree/Disagree/Agree/Strongly agree
9	All in all, I am inclined to feel that I am a failure.	Strongly disagree/Disagree/Agree/Strongly agree
10	I take a positive attitude toward myself.	Strongly disagree/Disagree/Agree/Strongly agree

## Results

Quantitative analysis

The present study enrolled 453 students with a mean age of 20 (±1.36) years (Range: 17-25 years). Table 3 shows the socio-demographic characteristics of the students. Out of 453 students, those belonging to the age group 20-22 years were 299 (66.00%), the age group 17-19 years were 121 (26.71%), and the age group 23-25years were 33 (7.29%). Males were 247 (54.53%) and females were 206 (45.47%). Among the students, the average age of males was 20 (±1.49) years whereas the average age of females was 20 years (±0.96). The students from the 1st phase were 142 (31.34%), the 2nd phase were 135 (29.80%), 3rd phase were 93 (20.53%) and 4th phase were 83 (18.83%). Among all, those who completed 12th from the state board were 350 (77.26%), Central Board of Secondary Education (CBSE) were 85 (18.77%) and Indian Certificate of Secondary Education (ICSE) were 18 (3.97%) students.

**Table 3:** Distribution of sociodemographic characteristics among study participants

Demographic Variables		Total Number of Students (N=453)	Percentage
Age in years	17-19	121	26.71
	20-22	299	66.00
	23-25	33	7.29
Sex	Male	247	54.53
	Female	206	45.47

Cyberbullying victimization

Among all participants, 154 (34%) were identified as victims of cyberbullying. Of these 92 (59.74%) were males and 62 (40.26%) were females (x2-2.56, p=0.1097, df-1). The most common form of cyberbullying victimization reported was ‘made fun of in chat room’ experienced by 234 (51%) students followed by upsetting posts on webpage reported by 113 (25%) (Table 4).

Cyberbullying offending

Among all participants, 95 (21%) were found to engage in cyberbullying others (offender). Among these, 87 (91.57%) were males and 8 (8.42%) were females (x2-66.68, p=0.0001, df-1). The most frequent offending behavior was reported to be sending hurtful computer or text messages, accepted by 142 (31%) students followed by posting things to make others angry or to make others laugh 114 (25%) students (Table 4).

**Table 4:** Prevalence and type of cyberbullying victimization and cyberbullying offending

Cyberbullying Victimization	Number of Student N=453 (%)
Been made fun of in a chat room	234 (51)
Received an email from someone you know that made you upset	68 (15)
Had something posted on social media that made you uncomfortable	85 (19)
Had someone posted something on another web page that made you upset or uncomfortable	113 (25)
Been bullied or picked on by another person online	92 (20)
Afraid to go on the computer	92 (20)
Something had been posted about you online that you did not want others to see	82 (18)
At least one of the above, 2 or more times	154 (34)
Cyberbullying Offending	
Posted something online about someone else to make others laugh	114 (25)
Sent someone a computer or text message to make them angry or to make fun of them	142 (31)
Posted something on someone’s Facebook, Instagram, or Snapchat page to make them angry or to make fun of them	71 (15.6)
Taken a picture of someone and posted it online without their permission	54 (11.9)
Sent someone an email to make them angry or to make fun of them	21 (4.64)
At least one of the above, 2 or more times	95 (21)

Table 5 indicates the distribution of self-esteem scores according to the type of cyberbullying (among victimized and cyberbullied students). Out of the 154 victims of cyberbullying, 110 (71.43%) had scores less than equal to 20 with a mean score of 14.63 (±1.12) indicating low self-esteem. And 44 (28.57%) victims had self-esteem scores above 20 with a mean score of 24.18 (±2.16) indicating high self-esteem. These students were able to cope with cyberbullying, maintaining their normal self-esteem. Out of the 95 offenders, a more even distribution was observed with 40 (42.1%) having self-esteem scores less than equal to 20, while 45 (57.9%) having scores more than 20. Out of 204 students who did not experience any type of cyberbullying, 59 (26.34%) reported lower self-esteem.

**Table 5:** Distribution of Students according to their self-esteem scores and type of cyberbullying (victims and offenders)

Types of Cyberbullying Experience	Self-esteem Scores	
	≤ 20 N (%)	>20 N (%)
Victims (N=154)	110 (71.43)	44 (28.57)
Offenders (N=95)	40 (42.1)	45 (57.9)
Never experienced (N=204)	59 (28.9)	145 (71.1)

Human psyche is complex and so is understanding self-esteem or one’s own worth. Table 6 depicts that, the cyberbullying victims had significantly lower scores than both, offenders (Z=3.313, p=0.001) and those who never experienced cyberbullying (Z=8.7651, p<0.0001). Additionally, Cyberbullying offenders also had significantly lower self-esteem than those who never experienced any kind of cyberbullying (Z=4.5521, p<0.0001).

**Table 6:** Self-esteem mean scores in cyberbullying victims, offenders, and those who never experienced cyberbullying

Types of Cyberbullying Experience	Self-Esteem Scores	Z-test, p-value
Victims (n=154)	19.41 ± 5.90	Z= 3.313, p=0.0011 for victims vs offenders
Offenders (n=95)	22.0 ± 6.14	Z= 4.5521, p<0.0001 for offenders vs never experienced cyberbullying
Never experienced cyberbullying (n=204)	26.44 ± 8.53	Z=8.7651, p<0.0001 for victims vs never experienced cyberbullying

Qualitative analysis

Thematic Analysis

The key themes emerging from FGDs were identified and analyzed based on the responses given by students in the discussions regarding their perception of cyberbullying, experiences, and solutions. The themes are as follows in Table 7.

**Table 7:** Thematic analysis of focused group discussion

Theme	Key components from FGDs (participants verbatim)	Reason/Interpretation/Solutions/Recommendations (Deeper insights/clarifications achieved through FGDs)
Understanding cyberbullying: Identifying key signs and behaviors	Online mental harassment was the word used by participants, where personal attacks are made via social media, such as blackmail, judgment, spreading false information, hate speech, insulting messages, exclusion from online groups, privacy violations (sharing personal info or photos without consent), and manipulated images. Spreading rumors, teasing appearance or abilities, deliberate exclusion, and posting private content without consent.	Interpretation: Students correctly identified and were aware of what cyberbullying is. The reason behind the occurrence of cyberbullying: A deliberate attempt by the offender to undermine the victim’s social status and personal dignity, focusing on exploiting perceived weaknesses.
Drivers of cyberbullying: Anonymity and personal motivation	Cyberbullies were often described as concealing their identity using fake profiles. Their motivations according to the participants, include jealousy, a desire for attention, revenge, and boosting self-esteem by exerting control over others, temporary self-satisfaction. Offenders also target individuals who have shared their personal vulnerabilities, making it easy to bully.	Reason for such offense: Offenders or bullies act out of a need to dominate or seek validation through harmful behaviors. Individuals who share their personal vulnerabilities are more likely to be bullied.
Short-term and long-term health impacts of cyberbullying	Short-term effects on health: Low self-confidence, stress, anxiety in social situations, decline in academic performance. Long-term effects on personality: Depression, suicidal thoughts, mistrust in relationships, lower academic success. Victims struggle with trust issues which largely affect their future relationships.	Inference: Recovery from cyberbullying and its consequences can be a long-term process, requiring continuous support and interventions.
Help-seeking and coping strategies: How the victims respond?	Emotional and social withdrawal of the victim is the most common coping mechanism. Immediate withdrawal from social media and social interactions delete accounts, block the bully victims hardly reach out to anyone for initial episodes but if cyberbullying happens repeatedly and is too bothering to them, they reach out first to friends/siblings or close family members and avoid disclosing the matter to the teachers or any authority in the institute. Victims do not want to seek help from police unless it is a financial fraud.	Inference: Emotional and social withdrawal once adopted as a coping mechanism, soon becomes a habit. Bitter experiences reduce sympathy and empathy traits and such persons become aloof or remain detached from relationships even in the future leading to long-term personality change. Reason for not seeking help in the first instance: Fear of being judged by friends and family; Reason for not seeking help at institute level: For the fear of academic repercussions, fear of being negatively judged by the teachers which stay for long or forever in teacher’s mind. Lack of trust in authority figures in the institute to address the issue sensitively. Reason for not seeking help from police: Victims fear reputation and social stigma attached once police are involved. Also, it is time-consuming with hardly anything to prove, they feel.
Practical approaches to deal with cyberbullying	Prevention and education study participants have suggested measures like “raising awareness” for identifying cyberbullying and coping mechanisms. Frequent discussion about such issues will normalize the topic and help reduce the stigma or fear attached. Measures to maintain or elevate self-esteem should be taught to students so that bullying does not cause distress. Dealing and coping with emotional disturbances which is also a life skill should be taught to students. A positive and supportive environment should be maintained around the victim.	Solution: Students need to be made aware of safe social media and online interactions, about cyberbullying and its hazards. Students should be encouraged to seek help by frequently addressing and discussing the issue breaking the barrier of fear, shame, and stigma. Topics like “Self-esteem”, “self-worth”, and “emotional intelligence” should become a part of the curriculum. Fostering a supportive environment at home and college.
Cyberbullying and institutional accountability: Steps toward effective interventions	Creating a special Cell/Unit or Department to deal with cyberbullying complaints. Students demanded that this should be a safe, non-judgmental space where students can report instances without fear of retaliation. Conducting awareness sessions and campaigns for cyberbullying. Counseling for both victims and cyberbullying offenders.	Inference: Cyberbullying is to be taken up as a serious issue and addressed appropriately at the institutional level. Institutional support is essential for not only addressing but also preventing and mitigating the effects of cyberbullying, ensuring both victims and bullies receive necessary help and interventions.

## Discussion

As digital platforms continue to evolve, both positive and negative impact of it on society is inevitable. In this study, the prevalence of cyberbullying victimization among medical students was 154 (34%), which was closer to the findings of two studies conducted in Gujarat by Parmar et al. [10] and Savani et al. [11] who observed a prevalence of 27.5% and 38.5%, respectively. Many other studies report a prevalence ranging from 15% to 35% [12-14].

The data suggested a strong relationship between self-esteem and involvement in cyberbullying. Specifically, cyberbullying victims had significantly lower self-esteem score (19.41±5.90) than both offenders (22 ± 6.14) and individuals who have never experienced cyberbullying (26.44± 8.53). This could imply that low self-esteem may make individuals more vulnerable to becoming victims of online harassment. Cyberbullying offenders also had lower self-esteem compared to those who have never been involved in cyberbullying, indicating that individuals with lower self-worth might engage in cyberbullying behaviors, possibly as a way to assert control or compensate for their own insecurities. Overall, individuals who never experienced cyberbullying had the highest self-esteem, suggesting that a positive self-image may act as a protective factor against becoming involved in or affected by cyberbullying. These findings highlight the potential psychological dynamics at play, where both victimization and offending are associated with lower self-esteem, though the relationship is more pronounced for victims. In FGDs, students confirmed that vulnerable individuals are targeted more by offenders in order to seek attention or revenge and gain temporary satisfaction. Students also agreed that cyberbullying not only had immediate effects like stress, and anxiety but also had long-term impacts on the personality such as inability to trust, low self-esteem, and depression.

In an effort to identify the motives of offenders behind cyberbullying, FGDs were conducted which revealed motives like jealousy, revenge, and a desire for attention or self-validation. It was noted that bullies may target individuals who have shared their personal vulnerabilities, emphasizing the need to dominate or control others. The idea of concealing identity using fake profiles also resonates with Hinduja and Patchin’s [14] argument about how anonymity emboldens individuals to engage in harmful behaviors without fear of repercussions. The current study suggests individual motivational factors whereas, Wang et al. [15] emphasized peer pressure and group dynamics as contributing factors in Taiwanese high school students. Both sources highlight that cyberbullying often arises from a desire for power and control, though. Establishing power is the desired outcome whether it arose out of individual motivation or peer pressure. Both sources agree that cyberbullying has a profound emotional impact on victims, leading to feelings of anger, helplessness, and isolation.

In this study, emotional and social withdrawal was identified as the most common coping strategies by victims of cyberbullying which resonates with the findings of Gupta et al. [16] and Gavcar et al. [17] who mentioned that the psychological distress and reduced self-esteem of victims lead to emotional and social disconnect to cope with the harassment. Both Gupta et al. [16] and Gavcar et al.’s [17] study findings align closely with the findings of the current study regarding the victim’s reluctance to seek help. Both studies shared the theme of fear of judgment from family, friends, and authority figures, along with a general lack of trust in institutions or legal systems to address the issue effectively. Fear of social stigma and academic repercussions are major barriers to help-seeking was reported in both studies corroborating the findings of the current study.

While examining the role of social support networks (family, friends, institutional resources, and system) in mitigating the effects of cyberbullying on self-esteem, the discussion with the medical students in this study revealed their need to have frequent discussions about such issues in order to normalize the topic and help reducing stigma or fear attached with it. Also, they wanted the institution to have separate cells to notify cyberbullying, to mitigate the effects by having counseling for victims and offenders, and to spread awareness regarding the same. Topics like emotional intelligence and self-esteem should be integrated into the curriculum as needed by the majority of students. The study by AL-Shaqsi et al. [18] emphasizes the role of cyberbullying awareness among students and the importance of coping strategies, such as emotional support from peers, developing self-esteem, and creating a safe space for victims similar to this study. However, students in the current study focused more on institutional role in case of safety, accountability, support, and resources with regards to cyberbullying. This may be so because the institution is the closest environment where students spend most of their time as compared to their homes and the institutions are in a better position to deal with legal implications and also can hold offenders accountable for their actions. Also, timely intervention by institutions can safeguard the reputation of the victims thus having an important role not only in dealing with cyberbullying but also preventing it.

Strengths, limitations, and further research

The study tackles a critical and underexplored issue like cyberbullying in medical undergraduates with validated instruments and qualitative insights into the experience of the victims and offenders of cyberbullying. To prevent stigma as victims or offenders, the scoring of the scales was not revealed to the students and FGDs were conducted from a general perspective. Hence, personal experiences as a victim or as a bully were not dealt with. This was arranged so as to prevent the triggering of hatred among students. Although the study highlights the immediate consequences of cyberbullying on self-esteem, study being a cross-sectional one, there is a lack of longitudinal research that tracks the long-term effects of cyberbullying over several years. Understanding how self-esteem and mental health evolve over time for victims could provide more comprehensive insights into the persistence of these issues. While some studies suggest that counseling, peer support and early training in emotional intelligence and resilience and self-awareness help mitigate the effects of cyberbullying, there is insufficient research on the comparative effectiveness of various interventions. Further research is needed to identify the most effective interventions.

## Conclusions

This research found the prevalence of cyberbullying about 154 (34%) and the victims of cyberbullying experienced a substantial decline in self-esteem 110 (71%), often leading to a range of psychological issues, including depression, anxiety, and social withdrawal leading to long-term consequences on an individual's self-worth and mental health. While support systems such as counseling and peer support have been shown to mitigate some of the adverse effects, the research suggests that greater efforts are needed from educational institutions to prevent cyberbullying and provide effective interventions. Raising awareness, better protective measures, and more resources for victims and integrating training in emotional intelligence and self-awareness are crucial to coping with cyberbullying and helping individuals regain their sense of self-worth and well-being.
